# Evidence for a Novel Marine Harmful Algal Bloom: Cyanotoxin (Microcystin) Transfer from Land to Sea Otters

**DOI:** 10.1371/journal.pone.0012576

**Published:** 2010-09-10

**Authors:** Melissa A. Miller, Raphael M. Kudela, Abdu Mekebri, Dave Crane, Stori C. Oates, M. Timothy Tinker, Michelle Staedler, Woutrina A. Miller, Sharon Toy-Choutka, Clare Dominik, Dane Hardin, Gregg Langlois, Michael Murray, Kim Ward, David A. Jessup

**Affiliations:** 1 Marine Wildlife Veterinary Care and Research Center, California Department of Fish and Game, Office of Spill Prevention and Response, Santa Cruz, California, United States of America; 2 Ocean Sciences Department, University of California Santa Cruz, Santa Cruz, California, United States of America; 3 Water Pollution Control Laboratory, California Department of Fish and Game, Office of Spill Prevention and Response, Rancho Cordova, California, United States of America; 4 Western Ecological Research Center, United States Geological Survey, Long Marine Laboratory, Santa Cruz, California, United States of America; 5 Monterey Bay Aquarium, Monterey, California, United States of America; 6 Department of Pathology, Microbiology and Immunology, School of Veterinary Medicine, University of California Davis, Davis, California, United States of America; 7 Applied Marine Sciences, Livermore, California, United States of America; 8 California Department of Public Health, Richmond, California, United States of America; 9 Division of Water Quality, State Water Resources Control Board, Sacramento, California, United States of America; Monash University, Australia

## Abstract

“Super-blooms” of cyanobacteria that produce potent and environmentally persistent biotoxins (microcystins) are an emerging global health issue in freshwater habitats. Monitoring of the marine environment for secondary impacts has been minimal, although microcystin-contaminated freshwater is known to be entering marine ecosystems. Here we confirm deaths of marine mammals from microcystin intoxication and provide evidence implicating land-sea flow with trophic transfer through marine invertebrates as the most likely route of exposure. This hypothesis was evaluated through environmental detection of potential freshwater and marine microcystin sources, sea otter necropsy with biochemical analysis of tissues and evaluation of bioaccumulation of freshwater microcystins by marine invertebrates. Ocean discharge of freshwater microcystins was confirmed for three nutrient-impaired rivers flowing into the Monterey Bay National Marine Sanctuary, and microcystin concentrations up to 2,900 ppm (2.9 million ppb) were detected in a freshwater lake and downstream tributaries to within 1 km of the ocean. Deaths of 21 southern sea otters, a federally listed threatened species, were linked to microcystin intoxication. Finally, farmed and free-living marine clams, mussels and oysters of species that are often consumed by sea otters and humans exhibited significant biomagnification (to 107 times ambient water levels) and slow depuration of freshwater cyanotoxins, suggesting a potentially serious environmental and public health threat that extends from the lowest trophic levels of nutrient-impaired freshwater habitat to apex marine predators. Microcystin-poisoned sea otters were commonly recovered near river mouths and harbors and contaminated marine bivalves were implicated as the most likely source of this potent hepatotoxin for wild otters. This is the first report of deaths of marine mammals due to cyanotoxins and confirms the existence of a novel class of marine “harmful algal bloom” in the Pacific coastal environment; that of hepatotoxic shellfish poisoning (HSP), suggesting that animals and humans are at risk from microcystin poisoning when consuming shellfish harvested at the land-sea interface.

## Introduction

During 2007, 11 dead and dying southern sea otters were recovered along the shore of Monterey Bay in central California with lesions suggestive of acute liver failure. Some animals were diffusely icteric and their livers were enlarged, bloody and friable. Expected causes for this condition, such as systemic bacterial infection were excluded via microscopic examination and diagnostic testing. Livers from affected animals tested positive for cyanotoxins (microcystins) via liquid chromatography-tandem mass spectrophotometry (LC-MS/MS) and hepatic lesions consistent with microcystin intoxication were observed microscopically. Environmental surveillance revealed that some local freshwater lakes and rivers supported *Microcystis* blooms during late summer and autumn, triggering the investigation reported here.

Cyanobacteria (formerly called “blue-green algae”) have a worldwide distribution and can form extensive blooms in freshwater and estuarine habitat. Toxin production by the cyanobacterium *Microcystis aeruginosa* was first reported in 1946 [1] and additional toxic species have been described. Exposure to environmentally stable microcystins in food, drinking water, nutritional supplements and during medical dialysis can cause significant and sometimes fatal hepatoxicity and possible tumor induction in humans and animals [2]-[6]. Microcystins are fast becoming a global health concern and recurrent blooms with toxin elaboration have been reported throughout Europe [7], [8], Asia [9], [10], Africa [11], [12], Australia [13], [14] and North and South America [15]–[17]. Factors that contribute to bloom formation and toxin production include warm water [18], [19], nutrient enrichment [19], [20] and seasonal increases in light intensity [2], [21]. Rising global temperatures and eutrophication may contribute to more frequent events and cyanobacterial “super-blooms”, with enhanced risks to human health [23].

Until recently, microcystin intoxication was considered a public health issue mainly of freshwater habitat, reflected by the vast body of published literature on potential human health risks due to microcystin exposure in rivers, lakes, reservoirs and freshwater aquaculture [4], [13], [16], [24], [25], [26]. In contrast, monitoring of marine water and seafood for similar risks has been limited, despite confirmation of outflows of microcystin-contaminated freshwater to the ocean [14], [17], [27], [28], detection of impacts by microcystins on copepods, corals and fish [29]–[31] and identification of proteins with protein phosphatase inhibitory activity in seawater, suggesting the existence of an additional class of marine “Harmful Algal Blooms” (HAB); hepatotoxic shellfish poisoning (HSP) [15].

The ability of potent and environmentally stable cyanotoxins to magnify trophically poses additional risks: Microcystin accumulation has been demonstrated in fresh and saltwater mussels [7], [32], farmed crustaceans [33], [34], fish [30] and possibly humans [26]. In addition, exposure of estuarine and marine biota to microcystins may trigger behavioral adaptations, such as decreased feeding on co-occurring nutritious species, that facilitate trophic transfer [9], [35]–[38]. Despite these concerns, worldwide shellfish sanitation and water safety programs do not typically include microcystin testing.

Along the Pacific coast of the United States, large-scale *Microcystis* blooms with toxin production occur each year in lakes and rivers throughout Washington [39], Oregon [4] and California [40], [41]. In California, *Microcystis*-contaminated runoff has been documented at the marine interfaces of the Klamath River [42] and San Francisco Bay [17]. Here we extend areas of concern to include the central California coast and document numerous marine mammal deaths due to microcystin intoxication.

The potential for cyanotoxins to flow to the ocean, resulting in deaths of marine species is a newly recognized problem. Demonstration of bioaccumulation in marine invertebrates and deaths of threatened southern sea otters due to microcystin intoxication provides strong evidence for significant and recurrent marine pollution by freshwater-derived microcystins within North America's largest national marine sanctuary. Because sea otters and humans utilize the same coastal habitat and share the same marine foods, our findings in sea otters are also likely to have important human health implications.

## Materials and Methods

Performance of this research was approved by the California Department of Fish and Game, Office of Spill Prevention and Response and the University of California.

### 1.) Environmental testing

Chemical confirmation of microcystin exposure in tissues from southern sea otters stranding during 2007 prompted investigation of local freshwater sources flowing into Monterey Bay for any prior history of cyanobacterial blooms. This investigation revealed that Pinto Lake, located approximately 8.5 km inland from Monterey Bay had a history of severe and recurrent *Microcystis* blooms with microcystin production. Visual examination of Pinto Lake, light microscopy and liquid chromatography-mass spectrophotometry/mass spectrophotometry (LC-MS/MS) testing in fall, 2007, confirmed the occurrence of an extensive *Microcystis* bloom with high toxin production, leading authorities to post warning signs at this location for several weeks. Stepwise sampling of water and surface bloom from Pinto Lake, its drainage into Corralitos Creek, and the Pajaro River that carries water from this region to Monterey Bay was performed during the bloom event.

Because bloom events are often ephemeral and patchy, sensitive methods are required to facilitate source tracking efforts. We investigated use of resin-based, Solid Phase Adsorption Toxin Tracking (SPATT) samplers to passively monitor fresh and salt water for microcystin contamination. SPATT was first proposed for HAB monitoring in 2004 to circumvent disadvantages associated with invertebrate bioassays [43]. To evaluate their performance under laboratory conditions, SPATT bags were placed into subsamples of concentrated water/*Microcystis* mixtures from Pinto Lake that were used for laboratory-based invertebrate exposures (section 3). Replicate SPATT samplers were also placed in each exposure tank during the invertebrate studies to assess consistency and repeatability of microcystin adsorption, and additional SPATT bags were deployed in the local marine environment and at the freshwater outflows of selected local rivers.

SPATT bags were constructed from 100 micron Nitex bolting cloth filled with 3 g (dry weight) HP20 (Diaon) resin. For activation, bags were soaked in 100% HPLC-grade methanol (MeOH) for 48 hours, rinsed thoroughly, transferred into a fresh volume of Milli-Q for MeOH residue removal by sonication and stored in Milli-Q at 4–6°C prior to use. Plastic embroidery hoops were used to fasten the bags in place during field deployment. After exposure, SPATT bags were evaluated using LC-MS for adsorption of domoic acid and microcystins as described below. In the laboratory, adsorption of environmentally-relevant concentrations of microcystins (hundreds of ppb) was observed in <24 h, following an exponential decay (adsorption) curve in a closed volume of filtered Pinto Lake water. In prior studies, 100% recovery of microcystin was achieved with simple extraction procedures (sequential 50% MeOH column extractions) (data not shown).

### 2.) Sea otter necropsy and microcystin testing

Sea otter carcasses were recovered by stranding network members, chilled with ice and transported to CDFG for necropsy as previously described [44]. Detailed postmortem examinations were performed by a veterinary pathologist and all major tissues were fixed in 10% neutral buffered formalin, trimmed, paraffin-embedded and 5 µm-thick, hematoxylin and eosin (H&E)-stained sections prepared and examined on a light microscope. Supplementary diagnostic testing included bacterial and fungal culture, immunofluorescence and PCR for common serovars of *Leptospira interrogans* and LC-MS/MS analysis of urine, gastrointestinal content, feces and urine for the presence of microcystin, domoic acid, okadaic acid, nodularin, yessotoxin and anatoxin-A. The primary and contributing cause(s) of death were determined based on gross lesions, histopathology and diagnostic results. Tissues, urine, serum and gastrointestinal contents were also cryoarchived at −80 C.

Analysis of water, tissue and digesta for microcystins was performed at the California Department of Fish and Game (CDFG) Water Pollution Control Laboratory or at the University of California, Santa Cruz. The preferred method of analysis post-extraction was high performance liquid chromatography tandem mass spectrometry (LC-MS/MS), following the protocols of Mekebri et al. [45]. Prior to testing, tissue samples were first homogenized using a Bucchi B-400 mixer equipped with a titanium knife assembly. Pre-weighed samples were mixed with methanol: water (90∶10) using a Polytron® homogenizer for four minutes, followed by sonication for one hour. The target analytes were microcystin (MCY)-RR, -Desmethyl RR, -LR, -Desmethyl LR, -LA, -LF, -LW, -LY and -YR, domoic acid, nodularin and okadaic acid. Certified calibration solution standards purchased from Sigma Aldrich and NRC-CNRC (Certified Reference Materials Program, Institute for Marine Biosciences, National Research Council of Canada) were used for method development, analyte identification and quantitation. HPLC-grade solvents (acetonitrile, methanol, water), glass fiber filters (Type A/E, 90 mm, 1 µm), Gelman Acrodisc® CR PTFE syringe filters (13 mm, 0.45 µm), and mobile phase additives, ACS grade formic acid (98%) and trifluoroacetic acid (99%) were also used. A combined intermediate MCY standard working solution was made in methanol and used to prepare a matrix spiking solution (20 ppb), which was serially diluted to develop a seven level calibration curve ranging from 0.2 to 200 ppb.

To determine total microcystin concentration and congener type(s) in water, the cyanobacterial cell walls were ruptured by repeated freeze-thawing and sonication and a 100 ml aliquot was filtered under vacuum through a glass fiber filter. Water and filters were extracted separately and filters containing planktonic material were extracted twice with 15 mL of methanol-acidified water (90∶10, v/v) by homogenizing for 1–2 minutes using a Polytron®, followed by 10 minutes of sonication in an ultrasonic bath.

For SPATT detection systems, only dissolved toxins were measured, so cell disruption was not required. Samples were analyzed using an Agilent 1200 liquid chromatograph (LC) connected to a 6130 quadrupole MS, using Selected Ion Monitoring (SIM). For all other samples, an Agilent 6410 triple quadrupole (QqQ) LC-MS was used for LC-MS/MS analysis. The following microcystin ions (m/z) were monitored: 519.8 -RR and 512.8 -desmethyl RR are both [M+2H]2+; 105.6 -YR, 995.7 -LR, 981.7 -desmethyl LR, 910.6 -LA, 1026.6 -LW, 987.6 -LF and 825.5 NOD-R were monitored using [M+H]+ using multiple reaction monitoring (MRM) mode. Full scan was also collected over the range 100–1100 amu. The MRM windows were established for microcystins using the MSMS product ions, which are the Adda fragments of m/z 135.2 and m/z 213 produced by the transition of the protonated parent ions. Agilent Mass Hunter software was used to collect and process data. The estimated method detection limits (MDL) and reporting limits (RL) for water samples were 0.02 µg/L (ppb) and 0.05 µg/L (ppb) for MCY and DA respectively, and 0.01 µg/L (ppb) and 0.02 µg/L (ppb) respectively for OA. The estimated method detection limit and reporting limit for tissues were 0.500 ng/g and 1.00 ng/g wet weight, respectively, for all toxins.

### 3.) Laboratory exposure of marine invertebrates

To assess microcystin uptake and retention by marine invertebrates consumed by humans and sea otters, freshwater/cyanobacterial mixtures were collected during a summer, 2009 *Microcystis* bloom at Pinto Lake. Dominance of *Microcystis* was confirmed microscopically and total microcystin concentrations were determined via LC-MS/MS. Live marine invertebrates were collected from Monterey Bay or purchased from commercial vendors, including species that are commonly farmed or harvested, such as Pacific oysters (*Crassostrea gigas*), manila clams (*Tapes semidecussatus*), mussels (*Mytilus edulis*), snails (*Tegula* spp.), red rock crabs (*Cancer productus*) and dungeness crabs (*Cancer magister*).

Three 1,022 L, temperature-controlled seawater tanks were used to complete the invertebrate exposure studies. The tanks were designed to permit water sampling at the top, middle and bottom of each tank, so that microcystin distributions could be followed through time and compared with results from invertebrate testing. Invertebrates were divided randomly between control (Tank 1), low exposure (Tank 2) and high exposure (Tank 3) tanks and allowed to acclimatize for 3 to 7 d. Bivalves and snails were placed in wire cages or plastic mesh bags and suspended at least 20 cm below the water surface. Large crabs were placed in plastic mesh enclosures that allowed them to range from just below the tank surface to just above the bottom. Snails were fed fresh *Macrocystis* kelp fronds and crabs were provided with chopped capelin (*Mallotus villosus*) every other day. Filter-feeders were exposed to plankton in continually flowing seawater from Monterey Bay until initiation of the microcystin exposure, and then from day 4 to day 21 of the experiment. Starting 4 days post-exposure, all tanks were continually flushed with clean seawater and water and invertebrate sampling continued for 21 days to determine post-exposure depuration characteristics for freshwater microcystins.

At the start of the exposures, a less concentrated *Microcystis* mixture collected from Pinto Lake during a bloom event (2.2 ppm [2,195 ppb] aqueous microcystin–LR mixed with suspended *Microcystis*) was added to the low exposure tank (Tank 2). A more concentrated mixture (10.6 ppm [10,600 ppb] aqueous microcystin–LR mixed with suspended *Microcystis*) was added to the high exposure tank (Tank 3) at the same time, while Tank 1 (the negative control) contained only seawater. Microcystin LR concentrations were measured in invertebrates and seawater sampled from the top, middle and bottom of each tank at regular intervals (24 H, 48 H, 72 H, 7 D, 14 D, and 21 D postexposure). During the first 96 H postexposure, invertebrates in tanks 2 and 3 were continually exposed to the microcystin-contaminated inoculum, while Tank 1 contained only recirculating, clean seawater and served as a negative control. Positive controls consisted of non-exposed water and invertebrate tissues spiked with known concentrations of a commercial preparation of microcystin-LR prior to LC-MS/MS testing.

Following 96 H of continuous microcystin exposure, all 3 tanks were flushed with clean seawater and sampling continued through 21d post-exposure. Prior to seawater flushing, water was collected from tanks 2 and 3, refrigerated and sub-sampled at the same intervals as invertebrates to determine persistence of microcystin toxin in seawater. Invertebrates were washed in tapwater prior to dissection to remove any surface contamination by *Microcystis* or microcystin. Invertebrate sub-sampling techniques reflected patterns of consumption by humans or otters: For snails, the entire body and shell was homogenized and tested whole, while the soft parts of bivalves and crabs were removed and the shells and carapace were discarded. The gastrointestinal tract and/or hepatopancreas was collected and screened for the presence of microcystin-LR in addition to archiving whole bodies and muscle tissue for evaluation as funds permit. All samples were refrigerated or frozen at −80 C prior to LC-MS/MS testing.

## Results

### 1.) Environmental testing

**Figure 1:**
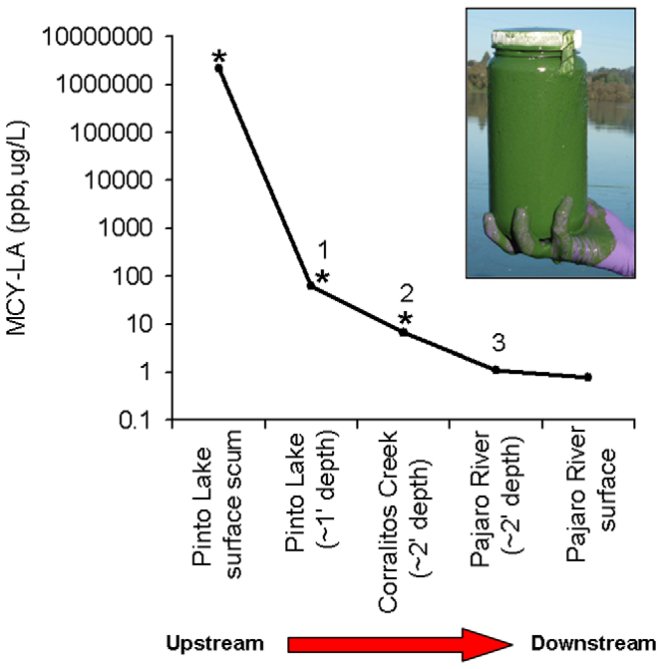
Tracing freshwater contamination by microcystins from land-to-sea. Inset: Sample of surface water collected during a “super-bloom” of *Microcystis* in Pinto Lake in fall, 2007 (Caution: Nitrile gloves and other appropriate personal protective equipment should be used to prevent dermal contact when collecting environmental samples of *Microcystis* and microcystins). Main figure: Time-matched microcystin-LA concentrations (ppb) in samples from Pinto Lake, just downstream in Corralitos Creek and the receiving waters of the Pajaro River within 1 km of Monterey Bay. Asterisks (*) indicate sampling locations where *Microcystis* was detected microscopically.

**Figure 2:**
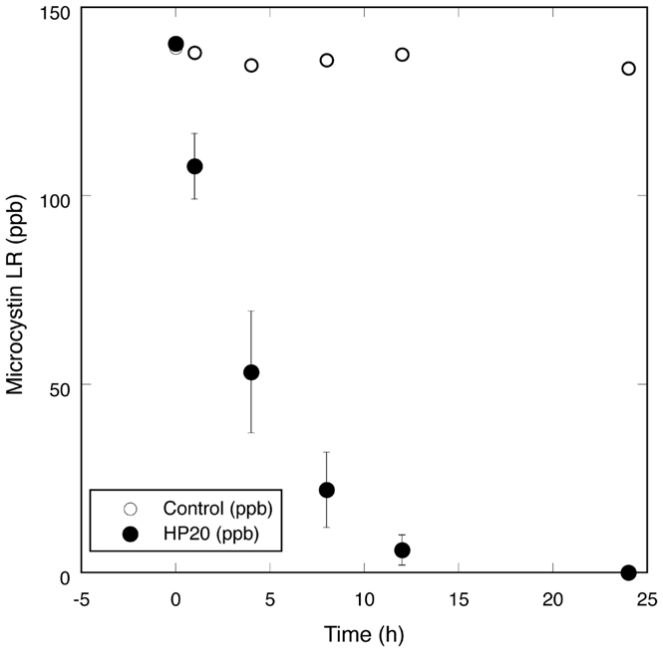
Evaluation of Solid Phase Adsorption Toxin Tracking (SPATT) sampler adsorption characteristics for freshwater microcystins. SPATT adsorption characteristics for microcystin were tested in the laboratory using Pinto Lake water amended with a known quantity of microcystin-LR. A control sample (open symbols) showed no change in microcystin concentration over time; In contrast, SPATT HP20 resin-based samplers (solid symbols: error bars represent standard deviation of 3 replicates) show rapid microcystin adsorption, with near-total depletion of microcystins from a controlled volume of water within <24 hours.

**Figure 3:**
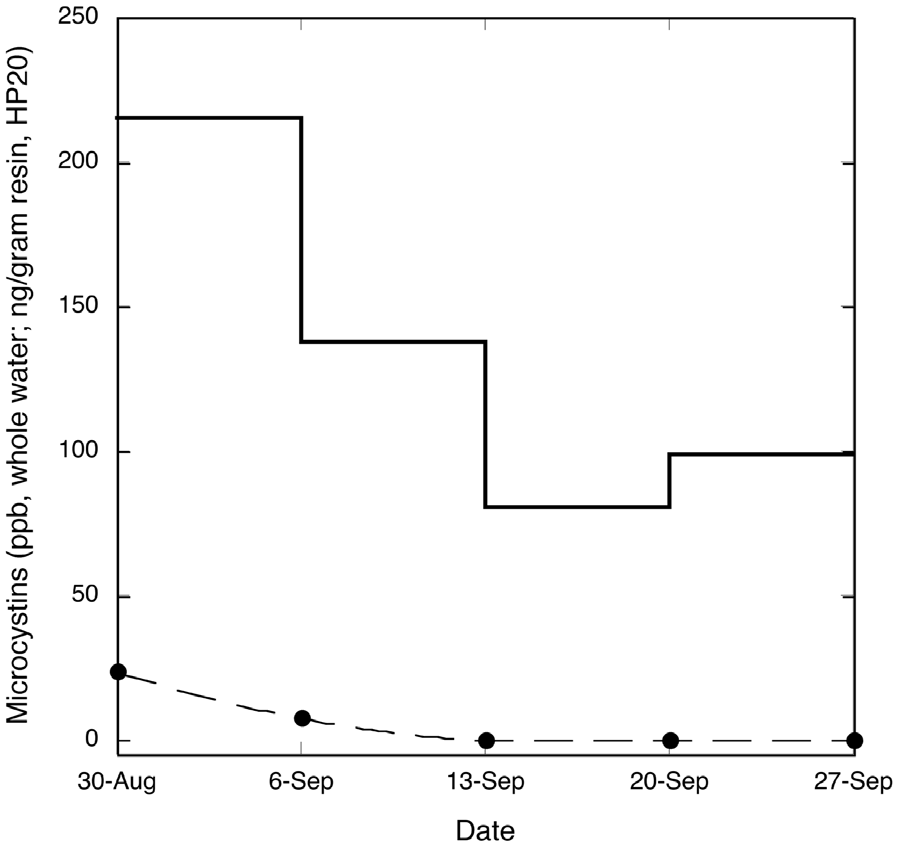
Variation in microcystin detection between conventional “grab” samples and Solid Phase Adsorption Toxin Tracking (SPATT). Comparison of microcystin (MCY-LR) detection in fresh water using intermittent “grab” sampling (sample periods indicated by black circles) and SPATT (solid line indicating weekly averaged toxin values) in Pinto Lake, demonstrating the higher sensitivity of SPATT for microcystin detection. Grab samples were collected at the beginning of each weekly SPATT deployment, and from the same sample location, so each 7-day integrated SPATT deployment is bracketed by two grab samples.

Analysis (LC-MS/MS) of water from Pinto Lake in fall, 2007 confirmed the occurrence of an extensive *Microcystis* bloom with high toxin production (Fig. 1). During this period, microcystin concentrations in scum from the surface of Pinto Lake exceeded 2,100 ppm (2.1 million ppb) MCY-LA, or approximately 2,900 ppm (2.9 million ppb) total microcystins, which is one of the highest microcystin concentrations ever reported from an environmental sample. During this same period, stepwise sampling of water and surface bloom from Pinto Lake, its drainage through Corralitos Creek and the Pajaro River confirmed the presence of *Microcystis* and microcystins from Pinto Lake to the river channel within 1 km of the ocean (Fig. 1). Recurrent *Microcystis* blooms with toxin production were also confirmed microscopically and via chemical analysis in samples from Pinto Lake and surrounding waters in 2008 and 2009 (data not shown). The most common microcystin congener that was detected in samples from Pinto Lake and the adjoining watershed during the 2007 event was MYC-LA, but MCY-RR, MCY-LR, MCY-Desmethyl-LR, MCY-LF and MCY-YR were also detected, and MCY-LR was repeatedly detected in Pinto Lake using SPATT between 2009 and 2010. No other biotoxins were detected in freshwater samples. Deployment of SPATT into microcystin/water mixtures collected from Pinto Lake demonstrated 100% adsorption of free MCY in <24 h (Fig. 2). In addition, higher sensitivity of SPATT for microcystin detection in water, when compared to intermittent “grab” samples, was also demonstrated (Fig. 3). During laboratory invertebrate studies, good agreement between duplicate SPATT bags suspended in each tank was noted for Tanks 2 and 3 (low and high microcystin exposure, respectively), while SPATT bags hung in Tank 1 (negative control) tested negative for microcystin (data not shown).

**Figure 4:**
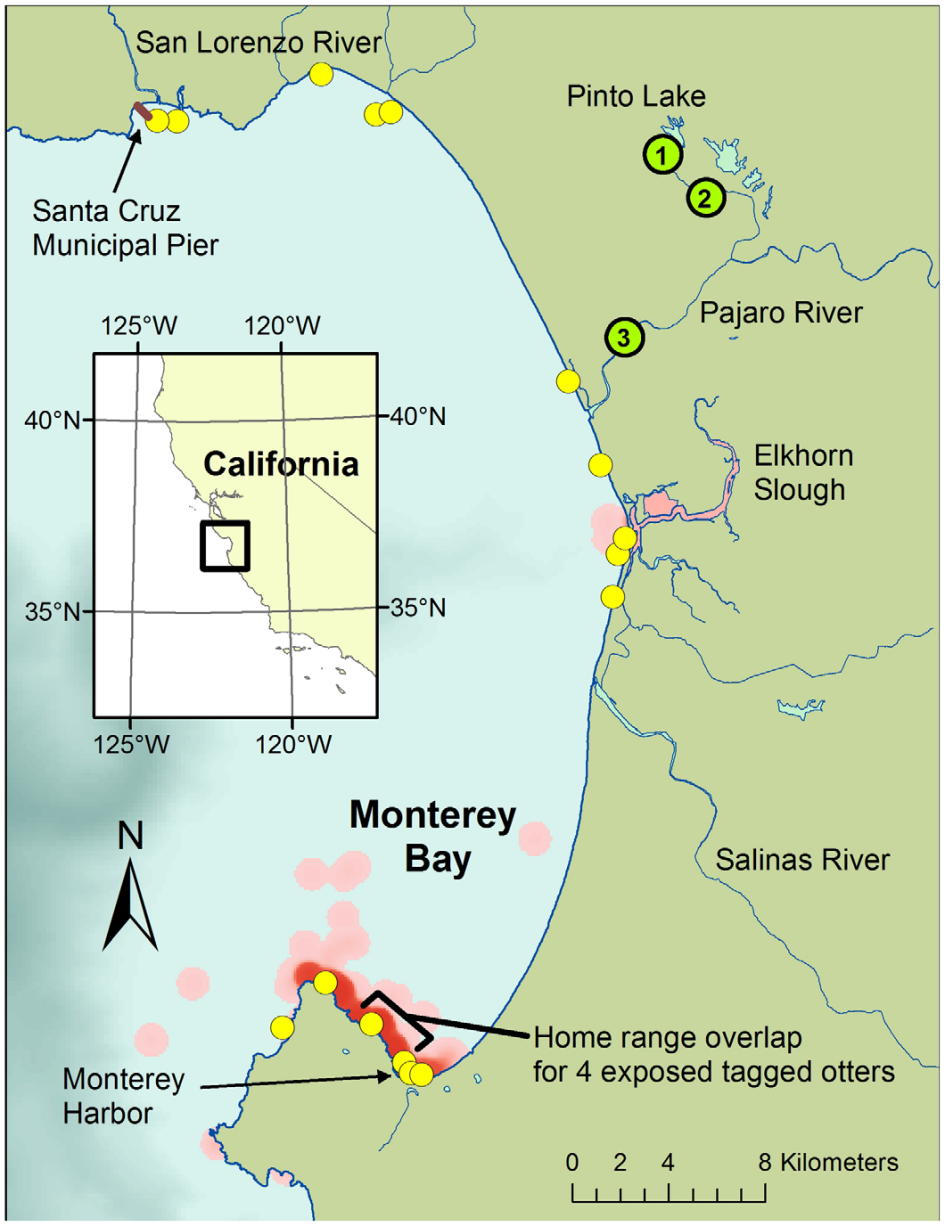
Map of Monterey Bay showing distribution of sea otters dying due to microcystin intoxication (yellow circles). Note spatial association of sea otter strandings with coastal locations of river mouths, harbors, coastal ponds and embayments. Habitat utilization distributions for 4 radio-tagged, microcystin-poisoned otters are plotted as kernel density distributions fit to daily re-sighting locations (red shading, with regions of most intense shading corresponding to the habitats most frequently utilized by affected animals). Locations of freshwater samples collected during a “Super-bloom” of *Microcystis* in 2007 are indicated by green circles, with numbers that correspond with the microcystin concentrations listed in Figure 1.

Using field-deployed SPATT, ocean water and the marine interfaces of selected coastal rivers flowing into Monterey Bay tested negative for microcystins during the dry season in summer and early fall of 2009, when freshwater runoff was minimal. However, at the onset of the fall rainy season (October-November, 2009), SPATT deployed at the marine outfalls of the Pajaro and Salinas Rivers tested positive for microcystins via LC-MS (data not shown). SPATT samplers deployed weekly in the ocean at the Santa Cruz Municipal Pier (Figure 4) during 2008 and 2009 routinely tested negative for microcystins, suggesting that the main source of these toxins for sea otters was not marine in origin. However, low levels of MCY-LR were detected at the pier (located near the mouth of the San Lorenzo River), after the first major storm event of Fall, 2009. Limited testing of fresh- and seawater and invertebrates from other regions of Monterey Bay did not reveal additional sources of microcystin-contamination (data not shown). Sea otter stranding patterns suggest that similar pollution events occurred in other coastal areas, but were not detected.

### 2.) Sea otter necropsy and microcystin testing

**Figure 5:**
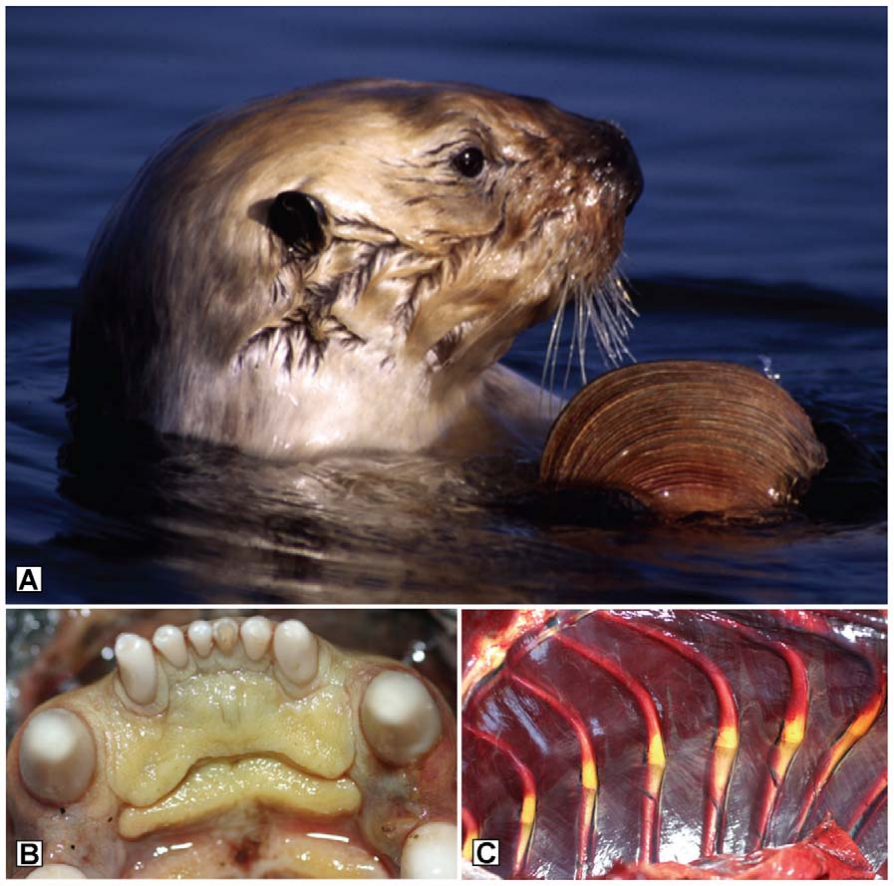
Microcystin detection in sea otter tissues was linked to bivalve consumption, liver damage and icterus. A.) Wild southern sea otter (*Enhydra lutris nerei*s) consuming a clam in Elkhorn Slough, Monterey Bay. B.) Diffuse icterus of oral mucous membranes of an otter poisoned by microcystin, due to severe hepatic damage and elevated plasma bilirubin. C.) Severe icterus of cartilage at the costochondral junction in a sea otter that died due to microcystin intoxication.

**Figure 6:**
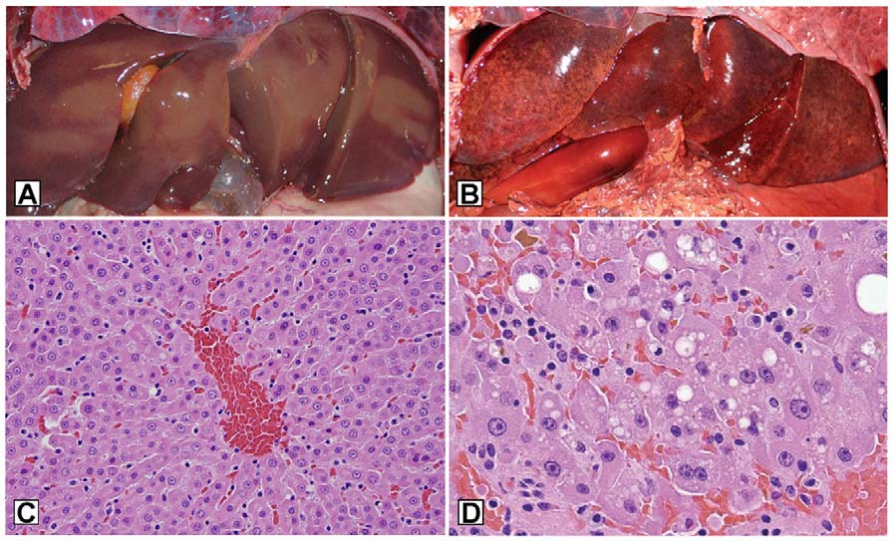
Gross and microscopic hepatic lesions of microcystin intoxication in sea otters, compared to control livers. A.) Gross appearance of normal sea otter liver. B.) Swollen, hemorrhagic liver from a sea otter that died due to microcystin intoxication. C.) Microscopic view of normal sea otter liver, D.) Microscopic appearance of liver from an otter that died due to microcystin intoxication, demonstrating hepatocyte swelling, cytoplasmic vacuolation, necrosis or apoptosis and parenchymal hemorrhage. Small greenish-gold accumulations of bile are apparent at the upper left and center-right portions of the photomicrograph.

Between 1999 and 2008, livers from 21 southern sea otters with gross and/or microscopic evidence of liver disease (Figs. 5 and 6) tested positive for microcystins via LC-MS/MS (Table 1). On the microscope, livers of microcystin-positive sea otters exhibited hepatocellular vacuolation, apoptosis, necrosis and hemorrhage (Fig. 6) consistent with previous descriptions of microcystin intoxication in humans and animals [1], [12], [13], [46], [13,47]. In contrast, livers from 2 captive sea otters (Table 1) and 19 wild otters without evidence of primary liver disease (data not shown) tested negative for microcystin. Carcasses of otters dying due to microcystin intoxication appeared to cluster near river mouths, coastal ponds, embayments and harbors, all areas with significant potential to receive and retain plumes of contaminated fresh water (Fig. 4).

**Table 1:** Stranding information and microcystin (MCY) concentrations (ppb wet weight) for wild, microcystin-positive sea otters and captive controls.

Animal number	Stranding date	Stranding region	Sample tested	MCY-RR	MCY-LR	MCY-Desmethyl LR
1280-04 (Captive control)	6/27/2002	N/A	Liver	nd^1^	nd	nd
1485-06 (Captive control)	11/14/2001	N/A	Liver	nd	nd	nd
3216-99	7/28/1999	Monterey Bay	Liver	1.36	nd	nd
3377-00	6/26/2000	Monterey Bay	Liver	2.04	nd	nd
3858-03	3/17/2003	Estero Bay	Liver	nd	11.8	nd
3955-03	5/8/2003	Monterey Bay	Liver	3.19	nd	nd
4240-04	6/5/2004	Monterey Bay	Liver	nd	nd	1.53
4294-04	8/25/2004	Monterey Bay	Liver	13.13	nd	nd
3110-98	5/14/2006	Monterey Bay	Liver	9.52	nd	nd
4811-06	8/25/2006	Estero Bay	Liver	7.71	nd	nd
4844-06	9/24/2006	Monterey Bay	Liver	3.62	nd	nd
4913-07	1/30/2007	Monterey Bay	Liver	61.58	nd	nd
5020-07	6/9/2007	Monterey Bay	Liver	38.45	348	nd
5023-07	6/9/2007	Monterey Bay	Liver	104.46	nd	nd
5036-07	6/25/2007	Monterey Bay	Liver	2.69	nd	nd
5082-07	8/16/2007	Monterey Bay	Liver	5.29	nd	nd
5108-07	9/23/2007	Monterey Bay	Liver	14.39	nd	nd
5167-07	11/21/2007	Monterey Bay	Liver & feces	18.7 & 16.4	nd	nd
5174-07	11/30/2007	Monterey Bay	Liver	6.18	nd	nd
5179-07	12/1/2007	Monterey Bay	Liver	3.76	nd	nd
5182-07B	12/2/2007	Monterey Bay	Liver	4.8	nd	nd
5185-07	12/6/2007	Estero Bay	Liver	1.97	nd	nd
5416-08	11/8/2008	Big Sur	Liver	7.58	nd	nd

1 nd  =  microcystin concentration was below minimum detection limits on liquid chromatography-tandem mass spectrophotometry.

The earliest confirmed case was in 1999 (Table 1). The greatest number of cases detected/year was in 2007, with 11 LC-MS/MS-confirmed sea otter deaths due to microcystin intoxication. Based on preliminary test results, 71% of known, microcystin-associated sea otter deaths have occurred since 2005 and 81% of affected animals stranded within Monterey Bay, California. However, additional cases were detected along the Big Sur and South-central California coastline, suggesting that multiple point-sources for microcystin exposure exist along the central California coast. One additional case was suspected based on lesions observed on histopathology, but the liver tested microcystin-negative, perhaps due to near-total loss of hepatocyte mass in the liver of this animal. Pathology associated with microcystin intoxication in sea otters will be described in greater detail in a subsequent manuscript.

Hepatic microcystin concentrations varied from 1.36 to 348 ppb wet weight (ww; Table 1). Various forms of microcystin (MCY-RR, -LR and -desmethyl LR) were detected in sea otter livers, with the majority of otters (19/21) testing positive for MCY-RR, compared to 2/21 and 1/21 for MCY-LR and MCY-desmethyl-LR, respectively. One liver tested positive for both MCY-RR and -LR. Feces of one otter recovered during necropsy also tested positive for microcystin, but it is unclear whether this represents ingestion of contaminated prey or enterohepatic toxin circulation. The microcystin congener that was detected at the highest concentration in a sea otter liver was MCY-LR (348 ppb ww). All water and tissue samples tested negative for okadaic acid, nodularin, yessotoxin and anatoxin-A. Urine from some otters with microcystin-positive livers also tested low-positive for domoic acid on LC-MS/MS; this biotoxin is widely distributed in sediments within Monterey Bay [48] and low levels of domoic acid are commonly detected in sea otter urine at necropsy (M. Miller, pers. commun.).

**Figure 7:**
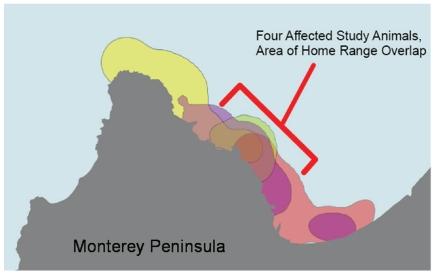
Overlapping home ranges of 4 tagged southern sea otters that died due to microcystin intoxication. Note the spatial overlap of all 4 home ranges on the north central Monterey Peninsula near Monterey Harbor (bracket): This harbor appears to be one of several high-risk locations for microcystin poisoning of sea otters, possibly due to prolonged retention of microcystin-contaminated water.

Four microcystin-positive otters had been previously captured and fitted with intraperitoneal VHF transmitters as part of long-term studies on habitat use, prey selection, and environmental exposure to biological pollutants. The home ranges of all four otters overlapped at the same point in south Monterey Bay (Fig. 7), suggesting a common source of microcystin exposure. Based on the spatial distribution of home ranges for radio-tagged southern sea otters [49], the likelihood of any four animals having overlapping home ranges by chance is <5.8%. Moreover, bivalve mollusks were the first or second most frequently consumed prey type for three of the four otters (for the fourth otter, no foraging data were available). This trait was shared with just 27% of all tagged animals; the majority preferentially fed on non-bivalve prey such as crabs, abalone or urchins [50]. Similar stranding clusters were identified for other microcystin-poisoned otters, leading to identification of several high-risk sites for microcystin intoxication along the shores of Monterey Bay (Fig. 4). Collectively these findings raised suspicion of bivalve prey as a possible vehicle for microcystin poisoning of sea otters.

### 3.) Laboratory exposure of marine invertebrates

Intact *Microcystis* cyanobacteria initially accumulated at the top of the seawater tanks, but were almost completely lysed after 48 hours. Microcystin–LR was detectable in seawater for the duration of the study (21 days) and microcystin concentrations in refrigerated seawater had declined only 44–71% from mean one hour post-exposure levels after 3 weeks (Table 2).

**Table 2:** Microcystin-LR concentrations (ppb) at the top, middle, and bottom of seawater tanks at two exposure concentrations (Tank 2 and Tank 3^1^) and varying postexposure intervals.

Inoculum 2,195 ppb	Tank 2 (low exposure)^1^					
	1 H	12 H	24 H	48 H	72 H	7 Days
Surface	2.34	0.87	5.18	0.75	1.32	Nd^2^
Middle	0.756	0.71	1.37	0.87	1.03	nd
Bottom	0.500	0.650	1.90	1.32	1.01	nd

Inoculum 10,600 ppb	Tank 3 (high exposure)					
	1 H	12 H	24 H	48 H	72 H	7 Days
Surface	32.8	5.88	22.2	4.02	6.58	nd^2^
Middle	5.53	5.01	7.46	6.09	4.95	nd
Bottom	5.03	5.03	7.66	3.69	6.75	nd
Mean		5.31	12.44	4.6	6.1	nd

1 All samples from Tank 1 (seawater control) tested negative for microcystin-LR. All 3 tanks were flushed with fresh seawater starting 96 H postexposure).

2 nd  =  microcystin concentration was below minimum detection limits on liquid chromatography-tandem mass spectrophotometry.

Significant bioconcentration of microcystin by marine bivalves (clams, mussels and oysters) and snails, but not large marine crabs, was documented (Table 3), with tissue concentrations of microcystin–LR up to 107 times higher in invertebrate tissues than in adjacent seawater. Microcystin concentrations in gastrointestinal tissues ranged from negative to 1,324 ppb wet weight (ww) in invertebrates, with the highest concentrations observed in clams, mussels and oysters sampled between 24 and 48 hours post-exposure.

**Table 3:** Microcystin LR concentrations (ppb wet weight) in marine invertebrate gastrointestinal tissues collected from Tank 3 (high microcystin exposure tank) at various time intervals post-exposure^1^.

Invertebrate Spp.^2^	24 Hour	48 Hour	72 Hour	7 Days	14 Days	21 Days
Manila clam	1,324	110	125	295	183	nd^3^
Mussel	979	3.13	14.3	45	64.4	30.5
Oyster	102	373	68.3	158	122	4
Dungeness crab	nd	nd	1.01	2.7	---	---
Red rock crab	nd	nd	nd	nd	nd	nd
Tegula snail	---	---	170	175	---	---
Seawater in tank^5^	12.4	4.6	6.1	nd	nd	nd

1 All tanks were flushed continually with clean seawater beginning at 96 H post-exposure.

2 n = 1 or 2 pooled invertebrates of each species at each sample point, except snails, where n = 7.

3 nd  =  microcystin concentration was below minimum detection limits on liquid chromatography-tandem mass spectrophotometry.

4 ---  =  not tested.

5 Average microcystin-LR concentration across the top, middle and bottom of Tank 3 at each time point.

Marine bivalves were also slow to depurate ingested microcystin; despite continuous seawater flushing beginning at 96 hours post-exposure, gastrointestinal microcystin concentrations at 14 days post-exposure were at 120%, 14% and 6.5% of 24 hour post-exposure concentrations for oysters, clams and mussels, respectively (Table 3). Mussel digestive tract remained microcystin-positive (30.5 ppb ww) 21 days after the initial exposure period and following 17 days of continuous exposure to clean seawater, which was the longest post-exposure timepoint evaluated during this study

Some variation in microcystin concentration between sample periods was attributed to differences in toxin uptake by individual bivalves, similar to the approximately 4-fold individual variability noted for bioaccumulation of domoic acid and saxitoxin in individual mussels from California coastal waters (R. Kudela, pers. commun.). All non-exposed controls (ambient seawater and invertebrates) tested negative for microcystin throughout the study (data not shown), which is consistent with a proposed freshwater (not marine) source of cyanotoxin exposure for sea otters.

## Discussion

Here we provide the first documentation of microcystin intoxication in a marine mammal. Our research confirms deaths of threatened southern sea otters from microcystin intoxication and incorporates 3 distinct, but interconnected lines of scientific inquiry to address the hypothesis that land-sea flow of microcystins with trophic transfer through marine invertebrates is the most likely route of sea otter exposure: 1) Time-integrative passive samplers were deployed in fresh and marine systems along the central California coast, confirming the presence of microcystins; 2) Necropsy, histopathology and chemical analysis of tissues from stranded southern sea otters and 3) Determining dynamics and persistence of freshwater microcystin uptake from contaminated seawater by marine invertebrates using controlled laboratory experiments. Although trophic transfer of biotoxins from planktonic species to higher vertebrates has been demonstrated for marine biotoxins like brevetoxin and domoic acid within estuarine and marine systems [51], [52], here we provide the first documentation of putative biotoxin transfer from the lowest trophic levels of nutrient-impaired freshwater habitat to top marine predators at the land-sea interface. Our findings provide the first hint of a serious environmental and public health threat that could negatively impact marine wildlife and humans.

We confirmed that Pinto Lake, a recreational water body located just inland from Monterey Bay exhibits substantial and recurrent *Microcystis* blooms. Biochemical testing of samples from Pinto lake during fall, 2007 revealed total microcystin concentrations of almost 2,900 ppm (2.9 million ppb), one of the highest microcystin concentrations ever reported from an environmental sample; the World Health Organization limit for microcystin contamination of finished drinking water is 1 ppb (0.001 ppm) [53]. Stepwise sampling of downstream tributaries during the late dry season confirmed the presence of *Microcystis* and microcystins throughout the lower Pajaro watershed to within 1 km of the ocean. Factors that facilitate development of cyanobacterial “super-blooms” in fresh water include elevated nutrient concentrations and salinity, warm temperatures, enhanced vertical stratification of lakes, summer droughts and increased light intensity; all factors that are exacerbated by global climate change [19], [22], [23], [54]. Cyanobacteria can exploit these conditions by developing intracellular gas vesicles and accumulating in dense surface blooms that “shade out” nontoxic phytoplankton like diatoms and green algae. They can also increase local water temperatures through light absorption, creating a positive feedback loop that helps ensure local dominance [23]. Once formed, these biotoxins can exert their effects in areas that are remote from sites of toxin production and can bioaccumulate in invertebrates and fish, suggesting a biologically plausible route for marine mammal (and human) exposure to freshwater toxins at the land-sea interface [32], [51], [52].

We demonstrated the excellent adsorption characteristics of SPATT resin-based systems for microcystin detection in both fresh and salt water. These passive samplers were more sensitive than periodic grab samples for field detection of microcystins and the ability to evaluate samples for the presence of multiple biotoxins simultaneously is an additional bonus. Preliminary environmental surveillance using SPATT revealed no detectable microcystin in nearshore marine waters of Monterey Bay until the fall rainy season, when the marine outfalls of the 3 most nutrient-impaired local waterways; the Salinas, the Pajaro and the San Lorenzo Rivers all tested microcystin-positive.

Because of their high metabolic rate, small home ranges and heavy reliance on nearshore-dwelling marine invertebrates as food, sea otters provide an upper trophic-level compliment to the SPATT resin for environmental detection of microcystins. Deaths due to cyanobacterial intoxication were first recognized in 2007, when 11 microcystin-poisoned sea otters were recovered along the shoreline of Monterey Bay. Microcystin intoxication appears to be an emerging health problem for southern sea otters. To date, at least 21 southern sea otters have died due to microcystin intoxication and the frequency of deaths may be increasing over time. Most microcystin-positive sea otters were recovered near embayments, harbors or river mouths. Sea otters generally do not venture into rivers to feed, so upstream exposure to microcystins is unlikely.

For radio-tagged otters that died due to microcystin intoxication, marine bivalves constituted a major portion of their diet. Sea otters routinely consume 25 to 30% of their body weight in clams, mussels, snails, crabs and other marine invertebrates daily [50], [55]. Marine bivalves are highly efficient biological filters for polluted water and can bioaccumulate a wide range of terrestrial-origin pollutants, including protozoa, enteric bacteria, viruses, biotoxins and anthropogenic chemicals [32], [56]–[58]. Embayments, harbors and river mouths are favored foraging sites, placing otters directly in the path of concentrated plumes of polluted water at the land-sea interface. A higher risk of exposure to terrestrial-origin pathogens and chemicals has been reported for sea otters residing near impaired habitats and those that feed preferentially on filter-feeding invertebrates [44], [49], [57], [59].

Microcystin accumulation has been demonstrated in fresh- and saltwater mussels, crustaceans, corals, fish and possibly humans [7], [30], [31], [32]. Our hypothesis that sea otters were most likely to be exposed to lethal levels of microcystins through consumption of contaminated invertebrate prey was evaluated through laboratory experiments where bioconcentration and depuration of freshwater microcystins by marine invertebrates could be assessed under defined conditions. We documented significant bioaccumulation and slow depuration of freshwater microcystins by marine oysters, clams, snails and mussels, with gastrointestinal tissue concentrations up to 107 times greater than adjacent seawater. Marine invertebrates were also slow to depurate ingested toxins, with high microcystin concentrations detected at 2 weeks post-exposure (Table 3). Freshwater microcystins were also relatively stable in seawater, with concentrations remaining at 29 to 56% of 1 hour postexposure concentrations, even after 21 days.

Collectively these data provide compelling evidence implicating land-sea flow with trophic transfer through marine invertebrates as the most likely route of biotoxin exposure. Detection of this problem initially in southern sea otters is not surprising, given the high level of scientific scrutiny of this federally-listed threatened species. Due to several unique aspects of their biology, including a high metabolic rate, a preference to feed near the shoreline and strong reliance on filter-feeding invertebrates as prey, southern sea otters have proven to be highly sensitive indicators of health of nearshore marine ecosystems [44], [49], [55], [60]. Southern sea otters are also a keystone species [61]; by foraging on kelp-feeding invertebrates like urchins, sea otters help maintain the complex 3 dimensional structure of the kelp forest that provides critical habitat for other marine wildlife [62].

Our data appear to strongly support the following hypotheses relevant to microcystin pollution and toxicity: H1: Significant concentrations of freshwater-derived microcystins are intermittently polluting the land-sea interface of central California. H2: These microcystins are causing mortality of threatened southern sea otters, possibly through trophic transfer to marine invertebrates feeding in contaminated freshwater plumes and H3: Wild and farmed marine bivalves consumed by sea otters and humans exhibit high microcystin uptake and slow depuration under conditions that mimic natural exposure. Monterey Bay, the region where the majority of microcystin-poisoned sea otters were recovered, forms the heart of the nation's largest marine sanctuary and is heavily utilized by humans for water contact recreation, tourism, fishing and wildlife viewing. No formal surveillance or regulatory system exists for microcystin detection in water or shellfish in most countries, including the United States. Because sea otters and humans consume many of the same marine foods, our research findings may reveal unrecognized health risks for humans when consuming invertebrates harvested at the land-sea interface.

## Acknowledgments

Sincere thanks go to Bryant Austin at Studio Cosmos and Robert Ketley at the City of Watsonville for contributing photos (Part A, Figure 1 and inset of Figure 3, respectively). We thank Jack Ames, Hannah Ban-Weiss, Francesca Batac, Gloria Blondina, Wayne Carmichael, Mary Curry, Erin Dodd, Traci Fink, Corrine Gibble, Dominic Gregorio, Michael Harris, Andy Johnson, Robert Ketley, Jenny Lane, Kamal Mekebri, Megan Olea, David Paradies, Meiling Roddam, Ben Weitzman and Karen Worcester for assistance with sample collection, invertebrate exposure and study design. We acknowledge the Monterey Bay Aquarium, the Marine Mammal Center, USGS-BRD, the United States Fish and Wildlife Service and CDFG for their efforts to recover and care for sick, stranded marine animals. Any use of trade, product, or firm names in this publication is for descriptive purposes only and does not imply endorsement by the U.S. Government or the State of California

## References

[1] Ashworth CT, Mason MF (1946). Observations on the pathological changes produced by a toxic substance present in blue-green algae (Microcystis aeruginosa).. Amer Jour Path.

[2] Jochimsen EM, Carmichael WW, An J, Cardo DM, Cookson ST (1998). Liver failure and death after exposure to microcystins at a hemodialysis center in Brazil.. N Engl J Med.

[3] Humpage AR, Falconer IR (1999). Microcystin-LR and liver tumor promotion: Effects on cytokinesis, ploidy, and apoptosis in cultured hepatocytes.. Environmental Toxicology.

[4] Gilroy DJ, Kauffman KW, Hall RA, Huang X, Chu FS (2000). Assessing Potential Health Risks from Microcystin Toxins in Blue-Green Algae Dietary Supplements.. Environmental Health Perspectives.

[5] Carmichael WW, Azevedo S, An JS, Molica RJ, Jochimsen EM (2001). Human Fatalities from Cyanobacteria: Chemical and Biological Evidence for Cyanotoxins.. Environmental Health Perspectives.

[6] Lankoff A, Carmichael WW, Grasman KA, Yuan M (2004). The uptake kinetics and immunotoxic effects of microcystin-LR in human and chicken peripheral blood lymphocytes in vitro.. Toxicology.

[7] Amorim Á, Vasconcelos V (1999). Dynamics of microcystins in the mussel Mytilus galloprovincialis.. Toxicon.

[8] Kohoutek J, Babica P, Bláha L, Maršálek B (2008). A novel approach for monitoring of cyanobacterial toxins: development and evaluation of the passive sampler for microcystins.. Analytical and Bioanalytical Chemistry.

[9] Hanazato T, Yasuno M (1987). Evaluation of Microcystis as food for zooplankton in a eutrophic lake.. Hydrobiologia.

[10] Ozawa K, Yokoyama A, Ishikawa K, Kumagai M, Watanabe MF (2003). Accumulation and depuration of microcystin produced by the cyanobacterium Microcystis in a freshwater snail.. Limnology.

[11] Oudra B, Loudiki M, Sbiyyaa B, Martins R, Vasconcelos V (2001). Isolation, characterization and quantification of microcystins (heptapeptide hepatotoxins) in Microcystis aeruginosa dominated bloom of Lalla Takerkoust lake-reservoir (Morocco).. Toxicon.

[12] Nasri H, El Herry S, Bouaicha N (2008). First reported case of turtle deaths during a toxic Microcystis spp. bloom in Lake Oubeira, Algeria.. Ecotoxicology and Environmental Safety.

[13] Dawson RM (1998). The toxicology of microcystins.. Toxicon.

[14] Robson BJ, Hamilton DP (2003). Summer flow event induces a cyanobacterial bloom in a seasonal Western Australian estuary.. Marine and Freshwater Research.

[15] Chen DZX, Boland MP, Smillie MA, Klix H, Ptak C (1993). Identification of protein phosphatase inhibitors of the microcystin class in the marine environment.. Toxicon.

[16] Domingos P, Rubim TK, Molica RJR, Azevedo SMFO, Carmichael WW (1999). First report of microcystin production by picoplanktonic cyanobacteria isolated from a northeast Brazilian drinking water supply.. Environmental Toxicology.

[17] Lehman PW, Boyer G, Hall C, Waller S, Gehrts K (2005). Distribution and toxicity of a new colonial Microcystis aeruginosa bloom in the San Francisco Bay Estuary, California.. Hydrobiologia.

[18] Zehnder A, Gorham PR (1960). Factors influencing the growth of Microcystis aeruginosa.. Canadian Jour Microbiol.

[19] Davis TW, Berry DL, Boyer GL, Gobler CJ (2009). The effects of temperature and nutrients on the growth and dynamics of toxic and non-toxic strains of Microcystis during cyanobacteria blooms.. Harmful Algae.

[20] Jacoby JM, Collier DC, Welch EB, Hardy FJ, Crayton M (2000). Environmental factors associated with a toxic bloom of Microcystis aeruginosa.. Canadian Journal of Fisheries and Aquatic Sciences.

[21] Tsuji K, Naito S, Kondo F, Ishikawa N, Watanabe MF (1994). Stability of microcystins from cyanobacteria: Effect of light on decomposition and isomerization.. Environmental Science and Technology.

[22] Welker M, Steinberg C (2000). Rates of Humic Substance Photosensitized Degradation of Microcystin-LR in Natural Waters.. Environmental Science & Technology.

[23] Paerl HW, Huisman J (2008). Climate: Blooms like it hot.. Science.

[24] Vasconcelos VM (1999). Cyanobacterial toxins in Portugal: Effects on aquatic animals and risk for human health.. Brazilian Journal of Medical and Biological Research.

[25] Chen J, Xie P, Zhang D, Ke Z, Yang H (2006). In situ studies on the bioaccumulation of microcystins in the phytoplanktivorous silver carp (Hypophthalmichthys molitrix) stocked in Lake Taihu with dense toxic Microcystis blooms.. Aquaculture.

[26] Backer L, Carmichael W, Kirkpatrick B, Williams C, Irvin M (2008). Recreational exposure to low concentrations of microcystins during an algal bloom in a small lake.. Marine Drugs.

[27] Matthiensen A, Beattie KA, Yunes JS, Kaya K, Codd GA (2000). [D-Leu^1^]Microcystin-LR, from the cyanobacterium Microcystis RST 9501 and from a Microcystis bloom in the Patos Lagoon estuary, Brazil.. Phytochemistry.

[28] Tonk L, Bosch K, Visser PM, Huisman J (2007). Salt tolerance of the harmful cyanobacterium Microcystis aeruginosa.. Aquatic Microbial Ecology.

[29] DeMott WR, Moxter F (1991). Foraging cyanobacteria by copepods: Responses to chemical defense and resource abundance.. Ecology.

[30] Malbrouck C, Kestemont P (2006). Effects of microcystins on fish.. Environmental Toxicology and Chemistry.

[31] Richardson LL, Sekar R, Myers JL, Gantar M, Voss JD (2007). The presence of the cyanobacterial toxin microcystin in black band disease of corals.. FEMS Microbiology Letters.

[32] Williams DE, Dawe SC, Kent ML, Andersen RJ, Craig M (1997). Bioaccumulation and clearance of microcystins from salt water mussels, Mytilus edulis, and in vivo evidence for covalently bound microcystins in mussel tissues.. Toxicon.

[33] Vasconcelos V, Oliveira S, Teles FO (2001). Impact of a toxic and a non-toxic strain of Microcystis aeruginosa on the crayfish Procambarus clarkii.. Toxicon.

[34] Zimba PV, Camus A, Allen EH, Burkholder JM (2006). Co-occurrence of white shrimp, Litopenaeus vannamei, mortalities and microcystin toxin in a southeastern USA shrimp facility.. Aquaculture.

[35] Fulton RS, III, Paerl HW (1987). Toxic and inhibitory effects of the blue-green alga Microcystis aeruginosa on herbivorous zooplankton.. J Plankton Res.

[36] Reinikainen M, Ketola M, Walls M (1994). Effects of the concentrations of toxic Microcystis aeruginosa and an alternative food on the survival of Daphnia pulex.. Limnology and Oceanography.

[37] Kotak BG, Zurawell RW, Prepas EE, Holmes CFB (1996). Microcystin-LR concentration in aquatic food web compartments from lakes of varying trophic status.. Canadian Journal of Fisheries and Aquatic Sciences.

[38] Ger KA, Teh SJ, Goldman CR (2009). Microcystin-LR toxicity on dominant copepods Eurytemora affinis and Pseudodiaptomus forbesi of the upper San Francisco Estuary.. Science of the Total Environment.

[39] Johnston BR, Jacoby JM (2003). Cyanobacterial toxicity and migration in a mesotrophic lake in western Washington, USA.. Hydrobiologia.

[40] Moisander PH, Lehman PW, Ochiai M, Corum S (2009a). Diversity of Microcystis aeruginosa in the Klamath River and San Francisco Bay delta, California, USA.. Aquatic Microbial Ecology.

[41] Moisander PH, Ochiai M, Lincoff A (2009b). Nutrient limitation of Microcystis aeruginosa in northern California Klamath River reservoirs.. Harmful Algae.

[42] Fetcho K (2008). http://www.klamathwaterquality.com/documents/2007YurokFINALBGAReport071708.pdf.

[43] MacKenzie L, Beuzenberg V, Holland P, McNabb P, Selwood A (2004). Solid phase adsorption toxin tracking (SPATT): a new monitoring tool that simulates the biotoxin contamination of filter feeding bivalves.. Toxicon.

[44] Miller MA, Gardner IA, Kreuder C, Paradies DM, Worcester KR (2002). Coastal freshwater runoff is a risk factor for Toxoplasma gondii infection of southern sea otters (Enhydra lutris nereis).. International Journal for Parasitology.

[45] Mekebri A, Blondina GJ, Crane DB (2009). Method validation of microcystins in water and tissue by enhanced liquid chromatography tandem mass spectrometry.. Journal of Chromatography A.

[46] Bishop CT, Anet EFLJ, Gorham PR (1959). Isolation and identification of the fast-death factor in Microcystis aeruginosa NRC-1.. Canadian Jour Biochem and Physiol.

[47] Fischer WJ, Dietrich DR (2000). Pathological and biochemical characterization of microcystin-induced hepatopancreas and kidney damage in carp (Cyprinus carpio).. Toxicology and Applied Pharmacology.

[48] Goldberg JD (2003). Domoic Acid in the Benthic Food Web of Monterey Bay, California [Master of Science]..

[49] Johnson CK, Tinker MT, Estes JA, Conrad PA, Staedler M (2009). Prey choice and habitat use drive sea otter pathogen exposure in a resource-limited coastal system.. Proceedings of the National Academy of Sciences.

[50] Tinker MT, Bentall G, Estes JA (2008). Food limitation leads to behavioral diversification and dietary specialization in sea otters.. Proceedings of the National Academy of Sciences.

[51] Scholin CA, Gulland F, Doucette GJ, Benson S, Busman M (2000). Mortality of sea lions along the central California coast linked to a toxic diatom bloom.. Nature.

[52] Flewelling LJ, Naar JP, Abbott JP, Baden DG, Barros NB (2005). Brevetoxicosis: Red tides and marine mammal mortalities.. Nature.

[53] WHO (1996). Cyanobacterial toxins: Microcystin-LR in Drinking Water..

[54] Guo L (2007). ECOLOGY: Doing battle with the green monster of Taihu Lake.. Science.

[55] Estes JA, Ray JC, Redford KH, Steneck RS, Berger J (2005). Carnivory and trophic connectivity in kelp forests.. Large Carnivores and the Conservation of Biodiversity.

[56] Miller W, Miller M, Gardner I, Atwill E, Byrne B (2006). Salmonella spp., Vibrio spp., Clostridium perfringens, and Plesiomonas shigelloides in Marine and Freshwater Invertebrates from Coastal California Ecosystems.. Microbial Ecology.

[57] Miller MA, Miller WA, Conrad PA, James ER, Melli AC (2008). Type X Toxoplasma gondii in a wild mussel and terrestrial carnivores from coastal California: New linkages between terrestrial mammals, runoff and toxoplasmosis of sea otters.. International Journal for Parasitology.

[58] O'Connor TP, Lauenstein GG (2006). Trends in chemical concentrations in mussels and oysters collected along the US coast: Update to 2003.. Marine Environmental Research.

[59] Miller MA, Byrne BA, Jang SS, Dodd EM, Dorfmeier E (2010). Enteric bacterial pathogen detection in southern sea otters (Enhydra lutris nereis) is associated with coastal urbanization and freshwater runoff.. Vet Res.

[60] Jessup DA, Miller MA, Kreuder-Johnson C, Conrad PA, Tinker MT (2007). Sea otters in a dirty ocean.. Journal of the American Veterinary Medical Association.

[61] Estes JA, Palmisano JF (1974). Sea Otters Their Role in Structuring Nearshore Communities.. Science.

[62] Estes JA, Danner EM, Doak DF, Konar B, Springer AM (2004). Complex trophic interactions in kelp forest ecosystems.. Bulletin of Marine Science.

